# Society Meetings

**Published:** 1905-01

**Authors:** 


### SOCIETY MEETINGS.

The Medical Society of the State of New York will hold its ninety-ninth annual meeting at Albany, January 31, February 1 and 2, 1905, under the presidency of Dr. Hamilton D. Wey, of Elmira. The program is being prepared under the direction of the business committee, consisting of Drs. Henry Flood, Elmira, chairman; A. E. Davis, New York, and L. H. Neuman, Albany. All who desire to participate in the scientific proceedings should address the chairman or other member of the committee.

The president of the American Medical Association, Dr. L. S. McMurtry, of Louisville, Ky., will attend the meeting and participate in the discussions.

---

THE Buffalo Academy of Medicine held meetings during the month of December, 1904, as follows:

*Section on Surgery.*—Tuesday evening, December 6. Program: (*a*) Prevention and treatment of shock and hemorrhage in surgical practice, George W. Crile, Cleveland; (*b*) The value of electrical conductivity of the blood and urine in diagnosis of kidney conditions, Harvey R. Gaylord.

*Section on Medicine.*—Tuesday evening, December 13. Program: (*a*) The dietetic use of predigested Legume flour, particularly in infants, D. L. Edsall, Philadelphia; discussion was opened by Irving M. Snow; (*b*) The etiology and symtomatology of cerebrospinal meningitis, with report of a case, Frank W. Love; (*c*) Report of a case of meningitis, Ray H. Johnston.

*Section on Pathology.*—Tuesday evening, December 20. Program: (*a*) A case of tuberculosis in the domestic turkey, C. A. Bentz; (*a*) Lymphosarcoma of the intestines, (*b*) Kidneys from cases of nephritis, considered in connection with urine examinations, N. G. Russell.

---

THE Medical Society of the County of Chautauqua held its semi-annual meeting at Dunkirk, Wednesday, December 14, 1904, under the presidency of Dr. W. J. French, Hamlet. The following program was observed:

Therapeutics, V. D. Bozovsky, Dunkirk; Typhoid fever, Joseph W. Reiger, Dunkirk; The treatment of retrodisplaced uteri, Herman E. Hayd, Buffalo; The physician and the milk supply, N. G. Richmond, Fredonia; Some foreign clinics, Charles A. Ellis, Sherman.

---

THE Erie County Medical Association held its quarterly meeting in Buffalo at the University Club, Monday, December 12, 1904, at 8.30 p. m. The program was: I. Presentation of cases and specimens; II. History of surgery in America, Roswell Park; III. Report of some cases, Vertner Kenerson.

The Medical Society of the County of Erie will hold its eighty-fourth annual meeting at the Buffalo Public Library building, Tuesday, January 10, 1905, at 10 a. m., under the presidency of Dr. William C. Krauss, Buffalo. The title of the presidential address is, The need of a medical library in Buffalo. The remainder of the program is in course of preparation.

